# Sleep deprivation affects gait control

**DOI:** 10.1038/s41598-021-00705-9

**Published:** 2021-10-26

**Authors:** Guilherme S. Umemura, João Pedro Pinho, Jacques Duysens, Hermano Igo Krebs, Arturo Forner-Cordero

**Affiliations:** 1 Biomechatronics Laboratory, Department of Mechatronics and Mechanical Systems of the Escola Politécnica, Universidade de São Paulo (USP), São Paulo, Brazil; 2 The 77 Lab, Department of Mechanical Engineering, Massachusetts Institute of Technology (MIT), Cambridge, MA USA; 3 Department of Neurology, School of Medicine, University of Maryland, Baltimore, MD USA; 4 Motor Control Laboratory, Movement Control and Neuroplasticity Research Group KU Leuven, Leuven, Belgium

**Keywords:** Sleep deprivation, Central pattern generators

## Abstract

Different levels of sleep restriction affect human performance in multiple aspects. However, it is unclear how sleep deprivation affects gait control. We applied a paced gait paradigm that included subliminal rhythm changes to analyze the effects of different sleep restriction levels (acute, chronic and control) on performance. Acute sleep deprivation (one night) group exhibited impaired performance in the sensorimotor synchronization gait protocol, such as a decrease in the Period Error between the footfalls and the auditory stimulus as well as missing more frequently the auditory cues. The group with chronic sleep restriction also underperformed when compared to the control group with a tendency to a late footfall with respect to the RAC sound. Our results suggest that partial or total sleep deprivation leads to a decrease in the performance in the sensorimotor control of gait. The superior performance of the chronic sleep group when compared to the acute group suggests that there is a compensatory mechanism that helps to improve motor performance.

## Introduction

Sleep disturbances affect cognition and lead to a decreased performance in learning, attentional and motor tasks^1^. There are many factors that can affect sleep. For instance, shift-workers are an extreme case of these socially imposed sleep impairment^2^ and they present a higher risk of suffering metabolic or cardiovascular problems, as well as attention and learning deficits^3,4^.

A common situation is shorter amount of sleep during workdays and compensation in free days, also known as social-jetlag^5^. This phenomenon may characterize a chronic sleep restriction and may cause a reduction in neurocognitive function^1^.

More than 85% of university students sleep less than 8 h per night and, among them, about 40% of the students sleep less than 6 h^6^. Therefore, most university students suffer from chronic sleep deprivation. Sleep deprivation affects negatively the learning process, attention and reaction time^7,8^. These impairments are associated with neural and cognitive diminished capacity related to lower metabolic rate in different regions of the central nervous system. Sleep deprivation is related to lower activity in the prefrontal cortex, thalamus, basal ganglia, and cerebellum areas associated with learning, cognition, motor control and sensory information processing^1,7,9,10^.

Human postural control has also been shown to be negatively affected by both acute sleep deprivation and chronic sleep restriction^11–13^. In contrast, the interaction between sleep disturbances and tasks such as gait has been less explored. Agmon et al.^14^ found that elderly people with impaired sleep quality performed worse under dual task condition. They reported an association between gait variability and speed with lower sleep quality, suggesting a compromised gait. Another study showed that college athletes with reduced sleep had greater difficulties in walking in tandem (toe-to-heel along a straight line) when executed together with a cognitive task. This was attributed to the deterioration of the executive functions caused by shorter sleep that resulted in a lack of attention^15^.

Gait is a rhythmic movement influenced by multifactorial components that are the result of the interaction of self-organized processes of neural and mechanical systems^9,16,17^. Several authors investigated the effects of an imposed external cueing via a metronome on human rhythmic motion, such as finger tapping or gait^18–21^. The sensorimotor synchronization with an exogenous pacing stimulus appears to be based on the prediction of the stimulus and anticipation of the motor outflow along with an error correction based on sensory information^22–27^.

In this respect, auditory motor synchronization involves several supraspinal structures such as the thalamus and prefrontal cortex, that can be affected by sleep deprivation^28,29^. The subliminal perturbations of the rhythmic auditory cueing—RAC^7,30,31^ represent changes that are below the threshold for explicit detection^19,28,32^, thus avoiding the changes in the level of alertness due to a perceived stimuli^33^, which is related to the sleep deprivation state of the subject. Therefore, the subliminal RAC changes could be used to “probe” the gait control system under sleep deprivation. We employed this unique paradigm that we previously developed^34^ to provide a basic RAC at a given frequency and then applied the changes of increasing and decreasing frequencies at very small increments (1 ms). The rhythm is maintained for a time interval before returning to the original RAC frequency. In this way, it is possible to test for potential after-effects.

Our goal is to identify changes in gait performance associated to sleep disturbances. We investigated the influence of different sleep schedules (control group with compensated sleep, chronic sleep restriction and acute sleep deprivation) on sensorimotor responses in a gait-auditory cueing synchronization paradigm with subliminal changes. We hypothesized that sleep disturbances affect the entrainment between gait and the RAC; therefore, we expected that the footfall and RAC would show larger divergences between the sleep deprived and the other groups, leading to larger errors and variability. Moreover, we expected to find that larger differences resulted in worse gait-auditory entrainment^35^.

## Methods

### Participants

A sample of young adults (35% female), college students, with no diagnosed motor, cognitive, sensory impairment or sleep disorders nor previous experience with our protocol, volunteered to participate in the experiment and signed an informed consent. The study was carried out in accordance with guidelines and regulations for experiments with humans and it was approved by the Ethics Committee of the University Hospital of the University of São Paulo and registered in the National database (Protocol number: 32626014.5.0000.0076).

The volunteers were instructed to follow their daily routines for two weeks while their rhythm and sleep were monitored by means of actigraphy. Two planned groups were allocated randomly, one group with n = 10 had one night of sleep deprivation before performing the gait tests (Sleep Acute Deprivation- SAD). When analyzing the sleep and rhythm parameters from the other group we found that there was a consistent mild sleep restriction and bad sleep quality for most participants, in agreement with previous literature about college students and sleep^6^. Within this sample, two different behaviors emerged: one sub-group compensated for loss sleep during the weekends and was considered as the reference Control Group (CG) and another that did not compensate over the weekend was named the Sleep Chronic Restriction group (SCR).

**Figure 1:**
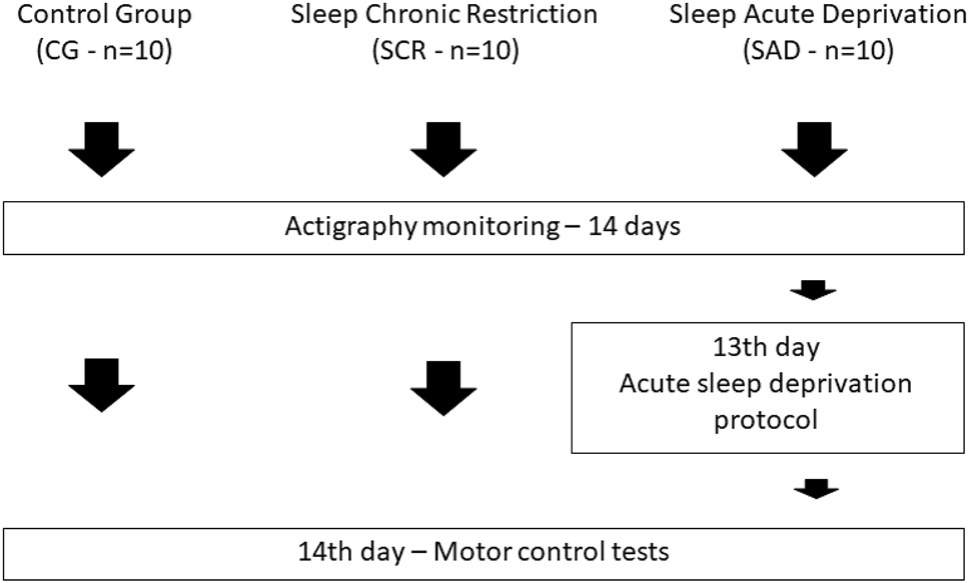
Experimental design used in this work. The sleep monitoring by actigraphy was conducted prior the motor control test.

The number of participants in GC and SCR groups were set to be equal to the number of SAD participants (Fig. 1): Control Group—CG (n = 10, 22 ± 2 years, 176 ± 7 cm, 72 ± 10 kg, 23.0 ± 1.9 kg/m^2^); Sleep Chronic Restriction—SCR (n = 10, 22 ± 2 years, 177 ± 8 cm, 73 ± 10 kg, 23.4 ± 1.8 kg/m^2^); and Sleep Acute Deprivation—SAD (n = 10, 22 ± 3 years, 166 ± 12 cm, 59 ± 16 kg, 21.0 ± 3.4 kg/m^2^). The control group is characterized by the sleep recovery on the weekends when compared with the weekdays. In the SCR group, there was no sleep recovery during the weekend (Table 1). The SAD group was composed of subjects that were subjected to one night of complete sleep deprivation.

**Table 1:** Mean and standard deviation of the sleep and circadian rhythm variables in the three groups prior to the experiment (CG, SCR and SAD).

Sleep and rhythm variables	CG	SCR	SAD	F ^*p-value*^
MEQ-HO (score)	43.30_(13.90)_	49.44_(10.74)_	46.30_(9.79)_	0.660 ^0.525^
PSQI (score)	5.00_(2.45)_	^**C**^ 8.20_(3.05)_	7.50_(1.72)_	4.656 ^**0.018**^
ESS (score)	9.50_(4.62)_	9.90_(3.31)_	^**CG, SCR**^ 20.00_(4.28)_	18.092 ^**<0.001**^
TST Workdays (hh:mm)	05:59_(00:53)_	06:21_(00:43)_	06:43_(01:30)_	1.084 ^0.354^
TST Free days (hh:mm)	^**SCR**^ 08:16_(01:12)_	06:26_(00:50)_	07:19_(01:47)_	4.592 ^**0.020**^
Sleep Efficiency (%)	0.89_(0.04)_	0.87_(0.04)_	0.89_(0.03)_	0.570 ^0.573^
IS (a.u.)	0.26_(0.07)_	0.21_(0.07)_	0.26_(0.08)_	1.350 ^0.278^
IV (a.u.)	0.75_(0.13)_	0.73_(0.17)_	0.71_(0.10)_	0.196 ^0.823^

*MEQ-HO* Morningness and Eveningness Questionnaire, *PSQI* Pittsburgh Sleep Quality Index, *ESS* Epworth Sleepiness Scale, *TST* Total Sleep Time, *IS* Inter-daily Stability, *IV* Intra-daily Variability. F value and p value of the one-way ANOVA test are presented in the last column. Holm–Sidak post-hoc results are expressed before the group means that yield statistically significant higher values.

### Study design

The volunteers wore an actimeter (ActTrust, Condor Instruments, Ltda, SP, Brazil) for 14 days before the gait experimental procedure. All gait tests were conducted on a Friday, starting after 8:30.

The participants in the SAD group came to the laboratory on a Thursday evening and stayed awake overnight. These subjects were instructed to stay awake until the next morning performing the same routine they would if they had to stay awake at their home. They could study, watch TV, play video games or read books. The consumption of caffeine or stimulants was not allowed.

### Sleep assessment

To control for individual sleep patterns, we assessed sleep and circadian rhythm with actimetry (ActiTrust, Condor Instruments Ltda, SP, Brazil). The actimeter collected movement data via a three-axis accelerometer at a sampling rate of 25 Hz, and recorded with a 60 s interval using a specific activity mode currently used in circadian and sleep monitoring research^36,37^. The ActStudio software (Condor Instruments Ltda, SP, Brazil) was used to interpret the actigraphy data. The following parameters were obtained: Sleep Efficiency (ratio between resting and sleeping time); Total Sleep Time; Time of Sleep Onset; Mid-Sleep Phase MSP (calculated using the actimeter variables total sleep time (TST) and sleep onset (SON), MSP = 0.5 ∗ TST + SON for work and free days); Inter daily Stability (circadian stability across multiple days); Intra daily Variability (the circadian variability along each day). The sleep parameters were calculated based on Cole-Kripke algorithm^38^.

Chronotype was assessed by the Morningness and Eveningness Questionnaire (MEQ-HO)^39^. This questionnaire provided information about the subjects’ routine preferences and it is associated with the time preferred for daily tasks, which can be associated with a performance peak in cognitive and motor tasks^40,41^. Higher values indicate evening chronotypes and lower values morning chronotype.

The Pittsburgh Sleep Quality Index (PSQI) was used to assess sleep disturbances and sleep quality. It has 19 items that provides information about sleep quality within the last 30 days. The sum of the components is used to indicate for possible sleep problems^42^. Higher PSQI values, i.e. bad sleep quality, are associated with actigraphy parameters, such as the L5, that is the amount of movement during the five hours of less motion^43^.

The Epworth Sleepiness Scale (ESS) was used to measure daytime sleepiness^44^. It is comprised of questions in which the participants self-report the chance of dozing off or fall asleep during day. Higher ESS indicate higher daytime sleepiness.

### Instruments

The gait experiments were performed on a motorized treadmill (Movement LX-160, Brudden, Brazil) and movement was captured by 6 infrared cameras with a sample frequency set at 120 Hz (Flex 13, Optitrack, Natural Point Inc., USA). These cameras captured 2 reflective markers placed on the participants’ heels. Motive software (Natural Point Inc., USA) was used to reconstruct those markers trajectories. A costume-made programmable RAC employing an Arduino Uno microcontroller (Arduino, Spa, Italy) was used to generate the beep sound through speakers along with digital LED pulses to synchronize the RAC and kinematic data.

### Familiarization trial

A familiarization period (5 min) with the treadmill at a fixed speed (set at 1.1 m/s) was given prior to the experimental protocol. The last minute of that period was recorded to assess the subjects’ self-selected gait period and step length without the RAC.

### Experimental gait paradigm

**Figure 2:**
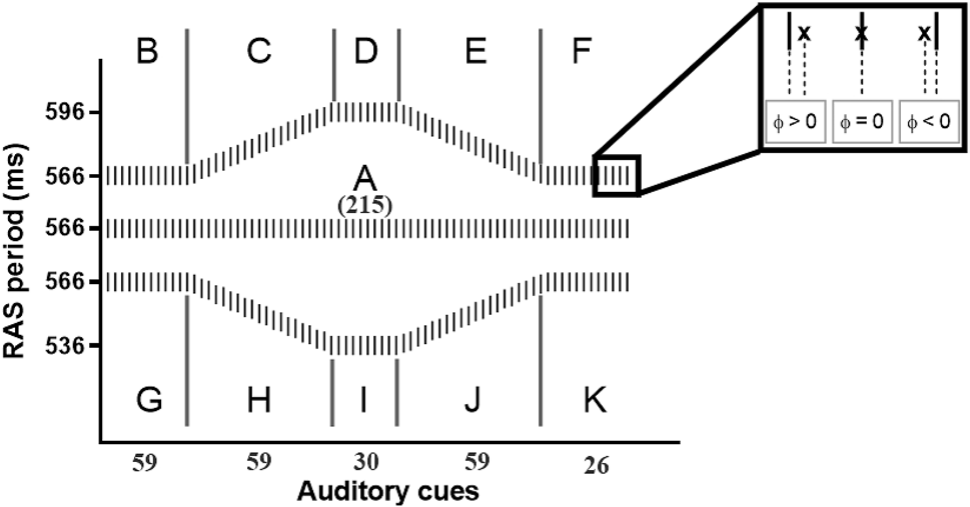
Eleven subphases of the three experimental conditions. The Relative Phase (ɸ) can assume zero if the footfall (x) coincides with the RAC sound (|), negative if it anticipates the sound and positive if it is delayed in respect to the sound. The numbers presented in the graph indicate the number of auditory cues in each phase ( Adapted from Forner-Cordero et al. 2019).

The experimental conditions consisted of three different RAC sequences: one constant (isochronous phase A) and two variable (non-isochronous: phases B-F and G-K) RAC conditions as shown in Fig. 2.

When the RAC frequency increases, the asynchrony between the stimulus and response decreases because the response is usually “advanced” with respect to the stimulus. On the other hand, when the RAC frequency decreases, the asynchrony increases^34^.

Before starting the tests, a verbal instruction was given for the participants to synchronize every footfall with the RAC sound, but they were not alerted to any modulation of the RAC beating. Participants were asked to perform each of the three experimental conditions presented in a random fashion until obtaining three trials for each condition. Period increments or decrements were of 1 ms ($\sim$ 0.6° relative phase), thus guaranteeing a subliminal stimuli variation as previously reported^34,45^. Since the treadmill velocity is constant, changes in the RAC frequency would change the step frequency, thus forcing a change in the step length to maintain the treadmill speed^46^.

In the first condition (A) the participants were asked to entrain to an isochronous stimulus (566 ms) for 215 steps^34^. In the second/third condition, they started with an isochronous stimulus (B/G), increase/decrease the stimulus period (C/H) until 596 ms/536 ms, assume new isochronous stimulus (D/I), to then decrease/increase the stimulus period (E/J) until 566 ms. The last phase was a new isochronous stimulus (F/K).

### Data processing

Custom-made algorithms were written in MATLAB (2015a, MathWorks, USA) to process the kinematic data. The reflective markers coordinates, after residual analysis, were filtered digitally by a low pass fourth order Butterworth filter with a cutoff of 12 Hz^47^. The heel strikes were determined by the shape of the foot markers trajectory using a cross-correlation function, as described in more detail elsewhere^48,49^.

To investigate whether subjects followed the rhythmic auditory cues, the synchronization error was calculated as a discrete Relative Phase angle (RP—Eq. 1):

1 $$RP=\frac{r\left(i\right)-s(i)}{T}\cdot 360;$$

where s(i) and r(i) are the stimulus (auditory cue) and response (heel strike) moment; and $\mathrm{T}$ is the RAC period. There are three possible outcomes: a negative Relative Phase (anticipation to the auditory cue), a positive Relative Phase (a delay) or zero (an accurate synchronization between footfall and cue). The absolute Relative Phase was used to compare the synchronization error between different phases because the sleep deprived group showed large positive and negative relative phases with a high variability and average values close to zero. For the analysis of the frequency of the accurate footfalls, a footfall was considered a hit when it occurred within a − 30 ms and 10 ms time-window from the beep, which are related to the values of the accuracy found in musicians and non-musicians in sensorimotor synchronization tests^50^. The footfalls taking place over 30 ms earlier than the beep were classified as early footfalls and the ones that occurred later than 10 ms were classified as late footfalls.

**Figure 3:**
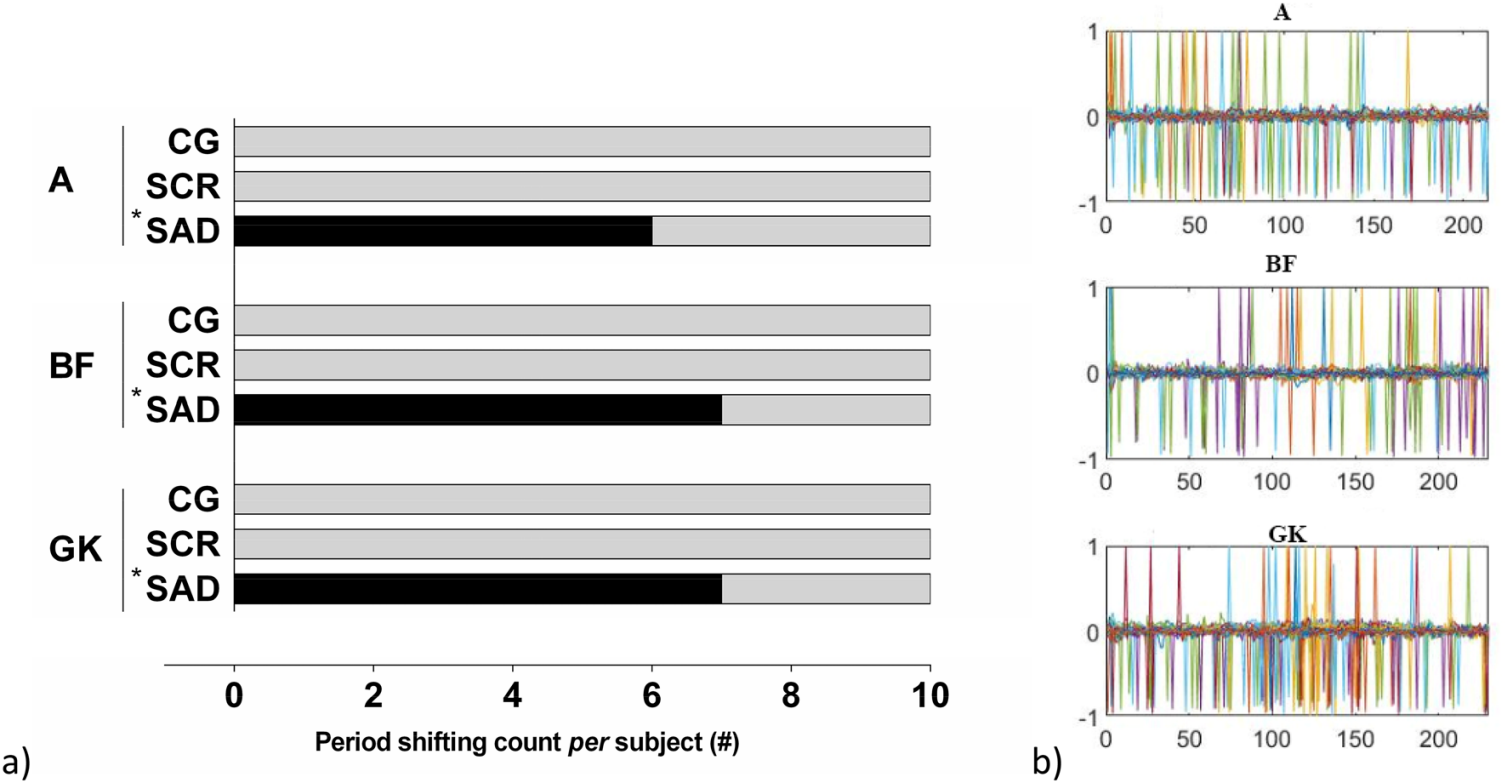
(**a**) Missed RAC sounds count per participant in the three experimental conditions. *Statistically different from the other groups yield by the Kruskal–Wallis test with post-hoc Tukey; (**b**) Missed beats (1 or − 1) of the RAC during more than 200 steps for the SAD group, where each color line represents one subject.

During the experiments, we noticed that several participants missed a few beeps. Those were recorded and shown in Fig. 3. We compared the between-group-differences of the missed RAC cues. To avoid introducing a systematic bias in the phase analysis, the missed cue was removed, and the Relative Phase was calculated with the closest footfall.

Secondly, we defined a parameter to measure the relation between RAC and step periods, the period error^51^. It was defined as the difference between step period and RAC period, in milliseconds, as shown in Eq. (2):

2 $$PE\left(i\right)=\left[r\left(i\right)-r\left(i-1\right)\right]-\left[s\left(i\right)-s\left(i-1\right)\right];$$

where r(i) represents the ith response (foot contact) to the ith stimulus (sound cue) s(i).

Step length was obtained by calculating the difference between the anterior–posterior forward heel coordinate and the rear one.

### Statistical procedures

All statistical procedures were conducted on SigmaPlot 11.0 (Systat Software Inc, Germany). The significance level for all tests was set at 5%. After visual inspection, Shapiro–Wilk, Levene and Mauchly tests were conducted to check for normality, homoscedasticity, and sphericity of the data, respectively. A two-tailed paired t test was used to compare ESS data in the SAD group between the day of the experiment and the day before. A one-way analysis of variance with pairwise multiple comparison procedures following Holm–Sidak method was used to compare the differences in sleep parameters between groups. Kruskal–Wallis test with Tukey post-hoc analysis was conducted to compare period-shift counts between groups. To compare the differences between groups and phases in step length, Relative Phase, and Period Error, we employed a two-way analysis of variance with Tukey post-hoc analysis. A Chi-Squared Test was conducted to compare differences between CG and SCR in the footfall accuracy. The Hedge’s g effect size was calculated for the comparison between CG and SCR or SAD groups at each phase of the test.

### Ethics approval and consent to participate

The experimental procedures were registered in the national database (Plataforma Brasil) and approved by the Ethical Committee of the Hospital Universitário da Universidade de São Paulo (CAAE: 32629414.9.0000.0076; CAAE: 32626014.5.0000.0076). All the subjects signed an informed consent.

### Consent for publication

 All the authors agree with the contents of the manuscript and this submission.

## Results

### Sleep and circadian parameters

Table 1 presents the differences between groups in all sleep and circadian rhythm variables. Statistical test results are also presented. The TST compensation was observed only in control group.

The comparison between ESS scores of SAD group the day of the experiment (M = 20.00, SD = 4.28, see Table 1) and the day before the experiment (M = 12.50, SD = 3.30, not shown in Table 1) indicates higher daytime sleepiness after the laboratory overnight, t(9)  = − 7.339, p ≤ 0.001.

### Accuracy of footfall in the gait task

As described previously, it was observed that subjects neglected some RAC sounds. Figure 3 presents the number of times the participants (per group) missed at least one auditory cue per trial for conditions with constant stimuli (phase A) or variable stimuli (phases B–F and phases G–K as explained in Fig. 2); along with the statistical differences between them.

**Figure 4:**
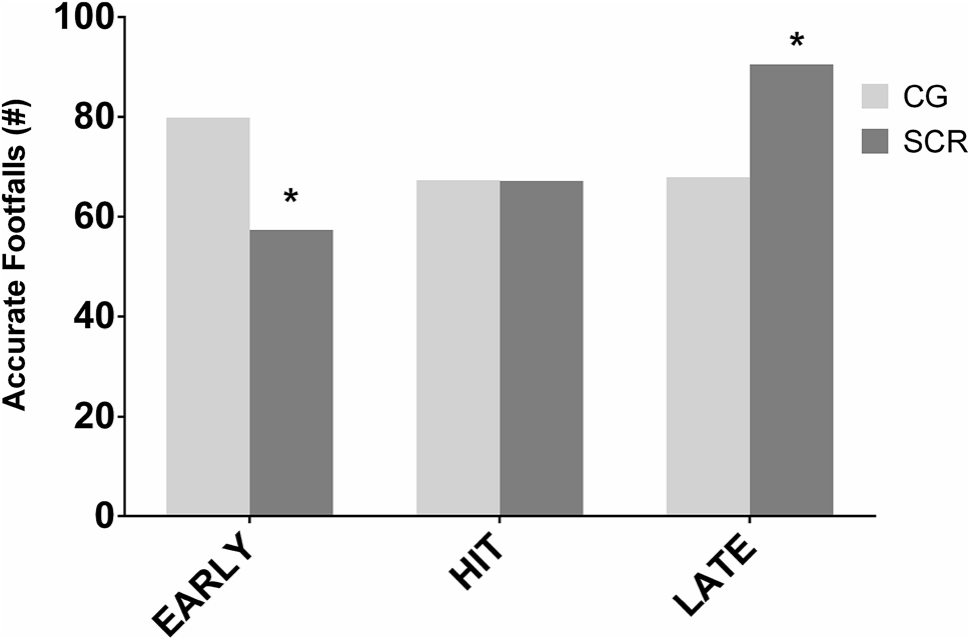
Number of accurate footfalls with respect to beep in the three possible situations early, late or hit for the CG and SCR groups in the isochronous condition (Phase A). *Indicates cross tabulation differences between groups in the Chi-Square Test.

The number of accurate footfalls is represented in Fig. 4 for phase A. For this variable, the Chi-Squared test showed a statistically significant differences (p < 0.05) between the CG and SCR groups in the frequency early (RP < -30 ms) and late footfalls (RP > 10 ms). Accuracy was less for SCR in early but better in late. No differences were observed between these groups for the hit interval (− 30 ms < RP > 10 ms).

### Gait synchronization with RAC

**Figure 5:**
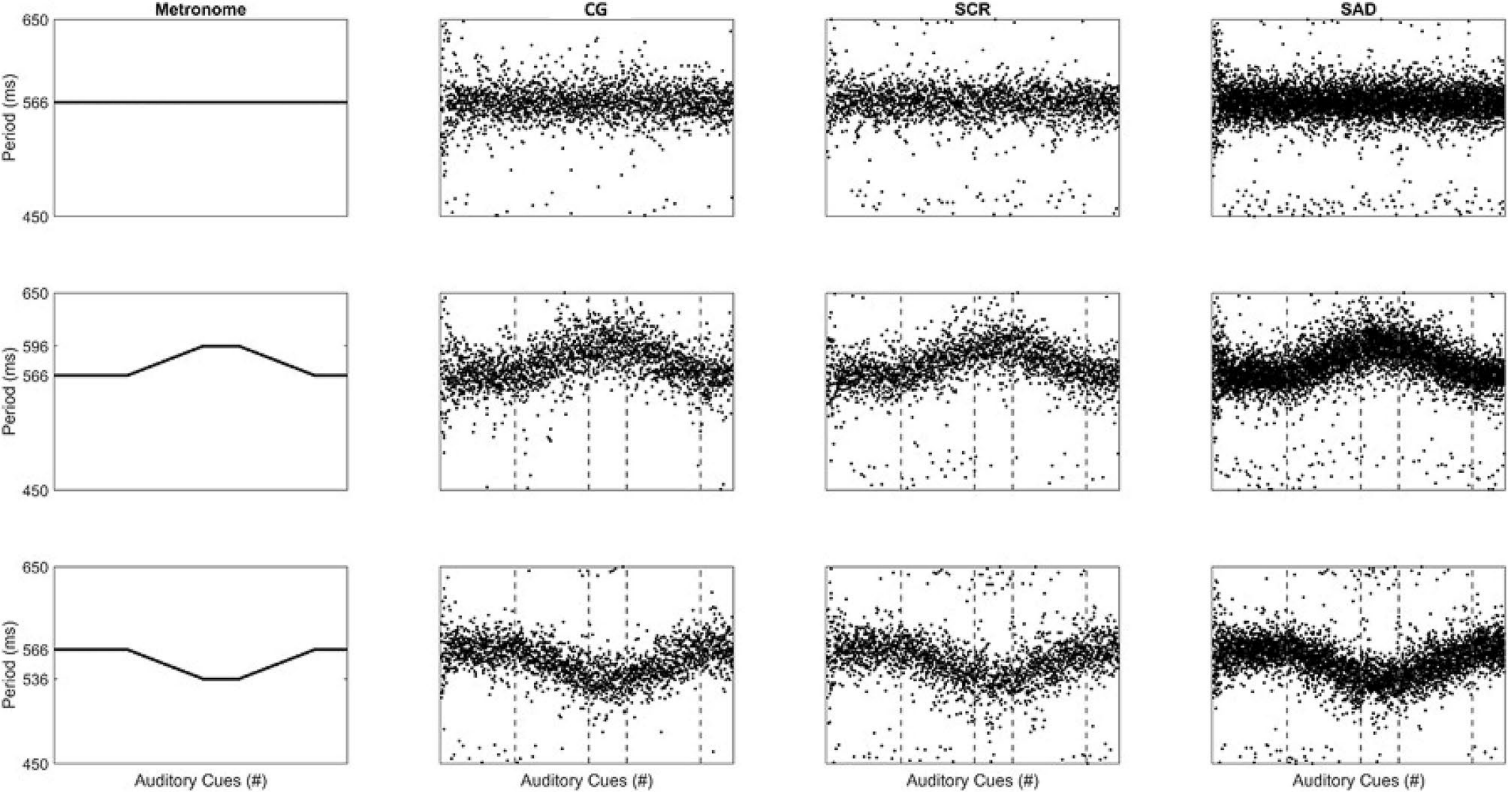
Overall step period data in one trial of all participants in the three groups in the three experimental conditions (first row: **A**; second row: **B–F**; and third row, **G–K**). First column expresses the RAC period. Dashed vertical lines represent the moment the RAC period changed.

The step period data for the three groups in the three experimental conditions is presented in Fig. 5. The mean and standard deviation for all gait variables are presented in Table 2 and the effects sizes between groups are shown in Table 3. Regarding Step Length, the ANOVA showed no statistically significant interaction between factors (F_22,297_ = 1.552, *p* = 0.057). A statistically significant main effect was found for Phase (F1_1,297_ = 61.240, *p* ≤ 0.001) and no significant main effect was found for Group (F_2,297_ = 1.060, *p* = 0.360).

**Table 2:** Mean and standard deviation of step length, step period, period error and the Relative Phase in the 3 experimental conditions (phases A to K) and in the no-cue condition (Ø – without RAC interference) in the three groups.

Variable	Group	Phase											
		Ø	A	B	C	D	E	F	G	H	I	J	K
Step length (cm)	CG	62_(4)_	63_(1)_	63_(1)_	64_(1)_	66_(1)_	65_(1)_	63_(1)_	63_(1)_	62_(1)_	60_(1)_	61_(1)_	63_(2)_
	SCR	62_(4)_	63_(2)_	63_(2)_	65_(1)_	66_(1)_	65_(1)_	63_(2)_	63_(1)_	61_(2)_	59_(3)_	61_(2)_	63_(2)_
	SAD	60_(4)_	62_(3)_	62_(4)_	62_(4)_	64_(4)_	64_(4)_	62_(3)_	62_(4)_	61_(3)_	59_(3)_	60_(3)_	62_(4)_
Step period (ms)	CG	553_(31)_	566_(1)_	567_(2)_	580_(1)_	596_(1)_	582_(1)_	566_(2)_	566_(2)_	552_(1)_	536_(2)_	550_(1)_	567_(2)_
	SCR	563_(25)_	566_(1)_	566_(2)_	580_(1)_	596_(2)_	583_(1)_	565_(2)_	566_(3)_	551_(1)_	535_(2)_	550_(1)_	565_(4)_
	SAD	541_(33)_	562_(9)_	561_(10)_	571_(13)_	581_(22)_	576_(14)_	562_(9)_	562_(10)_	550_(6)_	536_(5)_	548_(6)_	562_(11)_
Period error (ms)	CG	–	1_(0)_	2_(1)_	1_(1)_	2_(1)_	2_(1)_	2_(1)_	2_(1)_	1_(1)_	2_(1)_	1_(1)_	2_(3)_
	SCR	–	1_(0)_	2_(1)_	1_(1)_	1_(1)_	1_(1)_	2_(1)_	2_(1)_	1_(1)_	2_(2)_	1_(1)_	1_(2)_
	SAD	–	5_(9)_	6_(9)_	10_(13)_	15_(22)_	8_(12)_	5_(9)_	5_(9)_	4_(4)_	3_(4)_	4_(6)_	6_(9)_
Relative phase (°)	CG	–	28_(9)_	32_(16)_	38_(17)_	55_(21)_	37_(13)_	27_(12)_	29_(13)_	41_(23)_	52_(18)_	44_(22)_	39_(32)_
	SCR	–	30_(11)_	33_(20)_	35_(13)_	43_(15)_	29_(11)_	23_(10)_	24_(6)_	28_(6)_	35_(14)_	24_(7)_	26_(17)_
	SAD	–	52_(37)_	48_(29)_	62_(36)_	60_(34)_	54_(38)_	49_(37)_	41_(30)_	43_(32)_	42_(33)_	48_(36)_	50_(38)_

**Table 3:** Hedge’s g effect size between CG and SCR or SAD groups for step length, period error and relative phase, in each phase of the test.

Variable	Group	Phase											
		Ø	A	B	C	D	E	F	G	H	I	J	K
Step length (cm)	SCR	0.2 (− 0.68 − 1.08)	− 0.23 (− 1.11 − 0.65)	− 0.18 (− 1.06 − 0.7)	− 0.25 (− 1.12 − 0.63)	− 0.27 (− 1.16 − 0.61)	− 0.2 (− 1.08 − 0.67)	− 0.22 (− 1.1 − 0.66)	− 0.05 (− 0.93 − 0.83)	0.05 (− 0.83 − 0.92)	0.16 (− 0.72 − 1.04)	0.23 (− 0.65 − 1.11)	0.15 (− 0.72 − 1.03)
	SAD	− 0.42 (− 1.31 − 0.46)	− 0.49 (− 1.38 − 0.4)	− 0.47 (− 1.36 − 0.42)	− 0.23 (− 1.1 − 0.65)	− 0.1 (− 0.98 − 0.77)	− 0.6 (− 1.49 − 0.3)	− 0.41 (− 1.29 − 0.48)	− 0.59 (− 1.49 − 0.31)	− 0.76 (− 1.67 − 0.15)	− 0.76 (− 1.66 − 0.15)	− 0.38 (− 1.26 − 0.51)	− 0.26 (− 1.14 − 0.62)
Period error (ms)	SCR	–	− 0.23 (− 1.11 − 0.64)	0.31 (− 0.57 − 1.19)	0.06 (− 0.81 − 0.94)	− 0.75 (− 1.66 − 0.16)	− 0.74 (− 1.65 − 0.16)	− 0.01 (− 0.88 − 0.87)	− 0.35 (− 1.23 − 0.54)	0.34 (− 0.54 − 1.23)	− 0.02 (− 0.9 − 0.85)	0.03 (− 0.85 − 0.9)	− 0.24 (− 1.12 − 0.64)
	SAD	–	0.62 (− 0.28 − 1.52)	0.58 (− 0.32 − 1.47)	0.92 (0 − 1.84)	0.83 (− 0.08 − 1.74)	0.68 (− 0.22 − 1.58)	0.55 (− 0.34 − 1.44)	0.45 (− 0.44 − 1.34)	0.97 (0.04 − 1.89)	0.35 (− 0.53 − 1.23)	0.77 (− 0.14 − 1.68)	0.54 (− 0.35 − 1.43)
Relative phase (°)	SCR	–	0.15 (− 0.73 − 1.03)	0.05 (− 0.83 − 0.93)	− 0.21 (− 1.09 − 0.67)	− 0.67 (− 1.57 − 0.23)	− 0.63 (− 1.53 − 0.27)	− 0.38 (− 1.26 − 0.51)	− 0.43 (− 1.32 − 0.45)	− 0.69 (− 1.59 − 0.22)	− 1.08 (− 2.02 − − 0.14)	− 1.18 (− 2.13 − − 0.23)	− 0.48 (− 1.37 − 0.41)
	SAD	–	0.82 (− 0.09 − 1.74)	0.68 (− 0.23 − 1.58)	0.79 (− 0.12 − 1.7)	0.16 (− 0.72 − 1.03)	0.57 (− 0.32 − 1.46)	0.75 (− 0.15 − 1.66)	0.51 (− 0.38 − 1.4)	0.09 (− 0.78 − 0.97)	− 0.38 (− 1.26 − 0.51)	0.15 (− 0.73 − 1.03)	0.32 (− 0.56 − 1.2)

**Figure 6:**
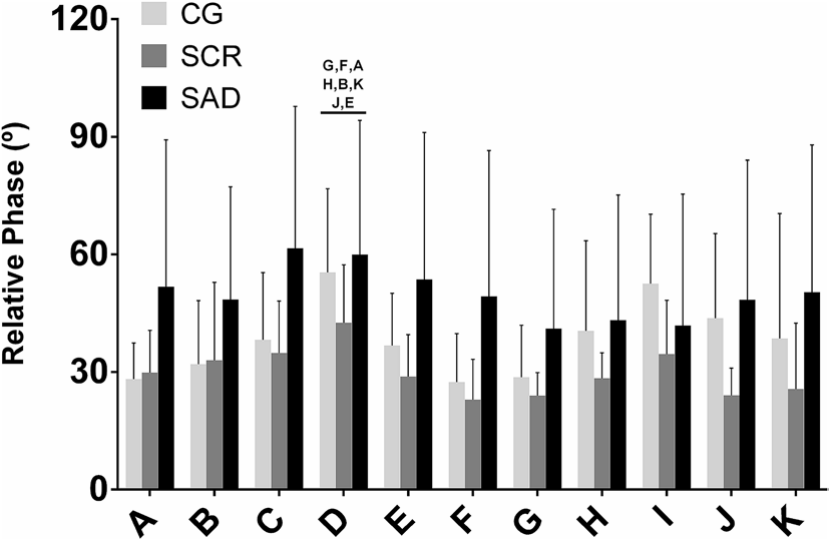
Footfall-RAC Relative Phase means and standard deviation in the three groups in the three experimental conditions defined by phase (**A**–**K**). The letters indicate the significant differences between phases. Note the higher relative phase and the higher variability in SAD group for Footfall-RAC Relative Phase in almost all experimental conditions. It is also possible to identify a trend for a synchronization error/RAC Period dependency.

Footfall-RAC Relative Phase did not show a statistically significant interaction between factors (F_20,270_ = 1.444, *p* = 0.102). Main effect Phase was found to be statistically significant (F_10,270_ = 4.439, *p* ≤ 0.001)—Fig. 6—and main effect Group was found to be non-significant (F_2,270_ = 2.787, *p* = 0.079).

**Figure 7:**
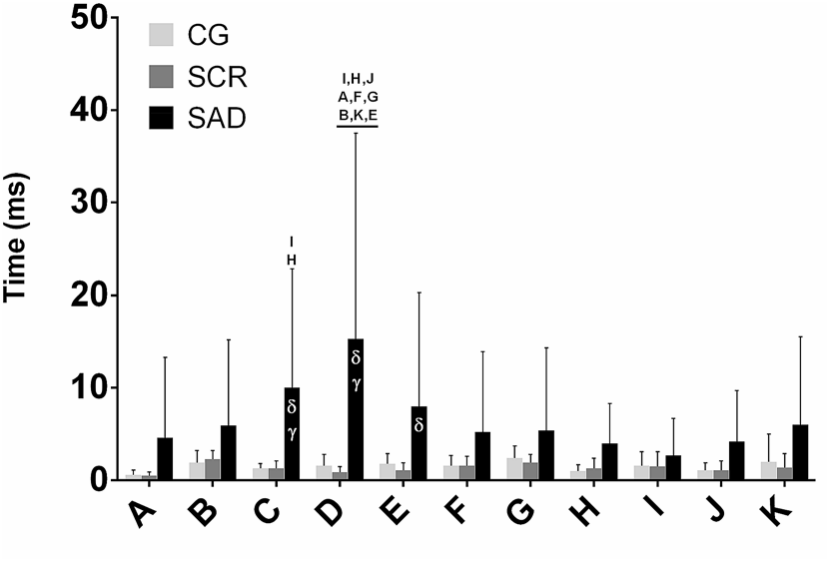
Means and standard deviation for Period Error in the three groups in the three experimental conditions for the various phases (**A**–**K**). Letters on top indicate main effect Phase differences and Greek symbols indicate main effect Group (δ: higher than SCR; gamma: higher than CG) in the two-way ANOVA. Note that low values mean better Period Error.

Regarding the Period Error (Fig. 7), a statistically significant interaction between factors (F_20,270_ = 2.663, *p* ≤ 0.001) was found. Main effect Phase was found to be statistically significant (F_10,270_ = 2.681, *p* = 0.004) as well as main effect Group (F_2,270_ = 3.373, *p* = 0.049).

## Discussion

We aimed to determine the effects of different sleep conditions on sensorimotor synchronization function to implicit subliminal changes in the rhythm of a RAC during gait. In a previous study^34^, we reported that individuals did follow these subliminal RAC changes. Here, we showed deleterious effects on gait control adjustments following rhythm changes in the group with acute sleep deprivation when compared to the control and chronic sleep restriction groups. Moreover, we also found that chronic sleep restriction, when not compensated during the free days, also impacted negatively on the Period Error.

### Relative phase and Period Error variations

Differences in Relative Phase between groups were stable across the eleven phases with the SAD group consistently showing the most important deviation, albeit not significantly different.

All groups were able to show a variation of the Relative Phase following the changes in the RAC rhythm. The Relative Phase is related to the feedback information to adjust gait. Phase angle between the auditory stimulus and the footfall generates information about accuracy, i.e., the ability to synchronize stimulus information and motor behavior. Larger variability in the Relative Phase values in the SAD group might explain the lack of interaction between groups in the analyses.

We found that control (CG) and chronic sleep restriction (SCR) groups had almost no Period Error through all the phases, while for the acute sleep deprived (SAD) group, the Period Error fluctuated across phases with values larger than zero and statistically significant different from the other two groups (see Fig. 7). This behavior suggests that the participants from the SAD group more frequently lost the synchronization with RAC. Note that if the Period Error presents values close to zero, it means that gait and RAC periods are similar, even if the footfall does not coincide precisely with the RAC, as assessed by the relative phase.

It has been proposed that the synchronization of gait with a rhythmic auditory stimulus occurs through two simultaneous processes: a prediction process (feedforward) of the sound cues—anticipatory adjustments to the next sound stimulus; and a feedback process—adjustments of the foot contact to match with the sound cue^34^. These two processes interact to control the gait rhythm and are in agreement with synchronization models proposed for the finger tapping tasks^22^.

The same model can explain present results. The Relative Phase accuracy seems to be related to a gait control process based on a feedback sensory loop driven by the difference between the sounds of the RAC and the footfall and leading to an automatic adjustment. Since no differences in Relative Phase were detected between groups, we can assume that the gait feedback control, driven by peripheral sensory inputs, was not significantly affected by sleep deprivation. In contrast, the period error of the gait pace to the RAC is the result of the matching of two cyclic events (RAC versus gait). This matching requires continuous attention, thereby relying more on cognitive resources.

The Period Error (difference between RAC and step periods) showed lower values in the CG and SCR groups. This good performance suggests a slow adaptation process based on a supraspinal oscillator that predicts foot contact concomitantly with the stimulus^22,34^. Even in this slower adaptation process, synchronization can be accomplished^52^. Higher Period Error in the SAD group suggests gait adjustments based on supraspinal processes likely affected by sleep deprivation.

The stimulus–response adjustments in sensorimotor synchronization have been traditionally studied in finger tapping experiments^23^. In these, a negative relative phase, indicating a prediction of the stimulus, the tap occurs before the sound cue^24^. This prediction could be based on a supraspinal internal oscillator that is used to predict the sound cue and elicit the motor action earlier to compensate for delays and reduce timing errors^53^. The RAC rhythm variations in finger tapping has been explained by two separate adaptive processes: phase and period corrections. The phase correction is sensory-based, does not require supraspinal adjustments, and is capable of keeping the movement in synchrony with the RAC with small timing perturbations^23,53^. However, with higher fluctuations in the rhythmic stimuli, this process is not able to keep synchronization^26^. The correction process adjusts the supraspinal oscillator period that controls the motor activity^53,54^. Some studies indicated that this correction in the period of the movement is under conscious and cognitive control, which requires attention and perception of the rhythm changes; thus, involving more cortical areas related to higher cognitive tasks^26,27,55^.

The processes of sensorimotor synchronization in finger tapping and rhythmic gait share similar neurobehavioral characteristics^20,34^. The prefrontal cortex, parietal lobe, basal ganglia, cerebellum and thalamus, are some of the brain areas responsible for finger tapping^23,56,57^ and are also activated while controlling gait^9^. Sleep disturbances are related to lower levels of metabolic activity in the thalamus, basal ganglia and cerebellum^58^, which have an important role in attention and real-time implicit adjustments of motor tasks^10,34,59^ and gait^9^. In addition, sleep deprivation strongly affects prefrontal cortex, a brain area associated with the ability to maintain attention, working memory and real-time movement control^60^.

Therefore, it was hypothesized that sleep deprived individuals would show a decreased performance in the period correction during our experiments. In this context, SAD participants used predominantly the feedback process (phase correction) for paced gait control. Moreover, most of the SAD group participants experienced a complete loss of synchronization with the RAC or one period shift in all trials of the three experimental conditions. In this case, there is a continuous delay/anticipation to the auditory cue, generating an increasing/decreasing positive/negative phase that accumulates through the test until it reaches the next/previous stimulus. In some subjects of the SAD group the phase errors were large. It seemed that they never fully synchronize with the RAC and with the implicit subliminal changes.

Footfall accuracy was characterized by the temporal difference between the auditory cue and footfall instants and a footfall was labelled as accurate when this difference was within a certain time window (− 30 to 10 ms). This time window was chosen based on experimental data on tapping experiments with different subject groups^50^. It was found that the SCR group showed a lower number of early footfalls in the isochronous rhythm condition (Phase A), when compared to the Control Group (Fig. 4). Moreover, the SCR showed a significantly higher number of late footfalls than the CG, but no differences was observed in the accurate hits. It is well known that, in sensorimotor synchronization tests, the subjects tend to anticipate the response. Therefore, it is possible to hypothesize that the feedforward gait control processes could be compromised in the SCR group, thus explaining the larger number of late footfalls^50,53,61^. The comparison with the SAD group was not made because the observation of the period shifting through the test (Fig. 3), which compromises the synchronization with the RAC during all the trial. The synchronization errors generate an excessive variation of the Relative Phase which compromise the validity of the analysis. However, the synchronization errors, represented by the phase shifts in Fig. 3 found in the SAD group provide sufficient evidence that the acute sleep deprivation affects the sensorimotor synchronization of the gait. Performance decrease in synchronization tasks is commonly observed in individuals with sleep deprivation^60,62^; and the inability to synchronize with the rhythm may be related to the lack of attention caused by sleep deprivation.

### Effects of sleep deprivation on sensorimotor-synchronization

Higher values of the Period Error in the SAD group can be explained by a decrease in alertness due to sleep deprivation^60^ or decreased activation in the basal ganglia and motor execution brain circuitry^10^. The SAD group individuals showed severe daytime sleepiness assessed by the ESS questionnaire. This condition is known to directly affect the cognitive functions^63^. The decrease in cognitive performance may result from attentional lapses, which are characterized by brief moments without behavioral response^7^. In general, these lapses are very brief, and it is not possible to detect them without a specific test. The Psychomotor Vigilance Test (PVT) successfully detects those attentional lapses based on delays in reaction time^64^. PVT lapses after 24 h of sleep deprivation are higher than in subjects without sleep deprivation, with the PVT lapses ranging from 4 to 10 in a 10 min test^65,66^. We were able to detect different levels of synchronization success with the RAC. We speculate that our experimental paradigm could be used to assess sleep disturbances, complementary to the PVT.

Decreased sleep efficiency and sleep quality are also aspects that can characterize chronic sleep restriction^67^. The SCR group had worse quality of sleep, as shown by the PSQI scores. Moreover, they did not compensate their sleep debt in the free days. Therefore, it is a group of individuals that are chronically sleep-restricted. Hence, we suggest that the difference in footfall accuracy found between CG, a group that compensated sleep debt during the free days, and SCR groups could be attributed to the uncompensated sleep debt.

In a previous study, we found that the sleep restriction in the workdays due to social jetlag impaired the balance control^11^. In the present study we found a decrease in the performance of a RAC paced gait task that depends on the sleep conditions of the participants. We have chosen to fix the step speed as well as the step period (with the metronome). However, there is a potential limitation in the use of a common speed for all the subjects. An alternative would weigh up a combination of preferred speed with preferred RAC period and set the frequency changes around this base frequency. Also, the leg dominance was not verified and should be recorded in future studies.

Nevertheless, despite of the limitations, in the acute sleep deprivation group, when the subjects did not sleep overnight, it was possible to detect gait control impairments when compared to the groups that were either chronically restricted or with a sleep restriction compensated during the free days. Therefore, it is possible to infer that higher sleep pressure leads to a performance decrease in a sensorimotor adaptation gait task. Although the best scenario is to avoid accumulating sleep deficit during the workdays, the compensation in the free days might be a suitable contingency strategy. Taking together, these results in gait control and implicit/procedural motor learning and adaptation might inform us on approaches to mitigate falls, particularly in the elderly.

## Conclusions

We analyzed a gait task of subjects with different sleep routines. The participants that had a one-night (acute) sleep deprivation had a worse performance in a gait sensorimotor task. Moreover, a control group (CG) of participants that compensated in their free days a sleep deficit accumulated in the workdays also showed better motor performance than the acute group. The results showed: (1) subliminal rhythmic compensation in gait is affected by sleep restriction and (2) sleep compensation results in a better motor performance.

## Data Availability

The experimental data are available.
